# Creating Non-Believed Memories for Recent Autobiographical Events

**DOI:** 10.1371/journal.pone.0032998

**Published:** 2012-03-09

**Authors:** Andrew Clark, Robert A. Nash, Gabrielle Fincham, Giuliana Mazzoni

**Affiliations:** 1 University of Hull, Hull, Yorkshire, United Kingdom; 2 University of Surrey, Guildford, Surrey, United Kingdom; Linkoping University, Sweden

## Abstract

A recent study showed that many people spontaneously report vivid memories of events that they do not believe to have occurred [1]. In the present experiment we tested for the first time whether, after powerful false memories have been created, debriefing might leave behind *nonbelieved memories* for the fake events. In Session 1 participants imitated simple actions, and in Session 2 they saw doctored video-recordings containing clips that falsely suggested they had performed additional (*fake*) actions. As in earlier studies, this procedure created powerful false memories. In Session 3, participants were debriefed and told that specific actions in the video were not truly performed. Beliefs and memories for all critical actions were tested before and after the debriefing. Results showed that debriefing undermined participants' beliefs in fake actions, but left behind residual memory-like content. These results indicate that debriefing can leave behind vivid false memories which are no longer believed, and thus we demonstrate for the first time that the memory of an event can be experimentally dissociated from the belief in the event's occurrence. These results also confirm that belief in and memory for an event can be independently-occurring constructs.

## Introduction

Counter-intuitive as it might sound, people do not always believe that the events they remember really occurred. Many people report having a memory that they know to be false [1], and in some cases these memories can concern extremely significant experiences. For instance, there are documented cases of people with memories of severe childhood abuse having encountered incontestable proof that the events they recall could not possibly have happened [2]. Here we report on an attempt to experimentally create *nonbelieved memories* in the lab by systematically stripping people's memories of their underlying beliefs.

Theoretical accounts of autobiographical memory and constructive memory processes have increasingly focused on believing as a foundation and precursor to remembering [3]–[5]. Scoboria, Mazzoni, Kirsch, and Relyea [6], for example, proposed a nested structure for autobiographical reasoning, whereby if an event is remembered then it will also be believed to have occurred, and if it is believed to have occurred, it will also be seen as plausible. Conversely, an event can be judged as plausible in the absence of belief, and can be believed to have occurred in the absence of a memory. This study [6] offered empirical support for this nested model: specifically, their participants gave ratings of plausibility, belief and memory for ten specific events that they might have experienced in childhood. The results showed that ratings were almost always higher for plausibility than for belief, which in turn was rated higher than memory. Indeed, participants gave belief ratings that were equal to or greater than their memory ratings on 95.7% of occasions.

It seems clear that the nested model provides a good account of the relationship between belief and memory. But what about the remaining 4.3% of occasions in Scoboria et al.'s study in which participants gave memory ratings that were higher than their belief ratings? Is this small percentage attributable merely to random error? It would appear not. In fact, there is both anecdotal and empirical evidence that nonbelieved memories do occur.

Perhaps the best-known anecdotal report of a nonbelieved memory was reported by Piaget [7], who vividly recalled being the victim of an attempted kidnapping in infancy. Thirteen years after this purported crime, Piaget learned that the whole event was a fiction fabricated by his nanny; yet Piaget maintained that he could still ‘remember’ it occurring. To explore incidences like Piaget's, Mazzoni et al. [1] recently reported the first empirical study of nonbelieved memories. The authors asked 1,593 undergraduates whether they could remember an event that they did not believe happened. Nearly a quarter of the sample reported having a memory of this type, thus establishing the status of nonbelieved memories as more than exceptional anecdotal oddities.

Mazzoni et al. [1] asked their participants about the characteristics of their nonbelieved memories, and found that these memories in fact had many phenomenological similarities with ‘regular’ believed memories. For example, both types of memory were rated similarly in terms of visual characteristics, emotional richness, and the feeling of ‘reliving’ and mental time-travel. Contrastingly, nonbelieved memories differed from believed memories on several other characteristics such as auditory quality and the sense of significance. These results led the authors to conclude that nonbelieved memories are experienced as genuine memories in many respects.

Mazzoni et al.'s [1] data are intriguing and informative, but to understand nonbelieved memories better, and thus to gain a stronger insight into the role of beliefs in memory construction, it would be beneficial to be able to create these memories experimentally. To consider how we might create nonbelieved memories, one should consider why people stop believing in their memories. Respondents gave numerous reasons, but the most common was that someone else informed them that the event did not occur [1]. Similarly, in studies of co-witness influences upon memory, participants who remember particular details are often far less likely to privately report those memories after they receive feedback from a confederate denying the presence of those details [8]. An analogous process to this occurs after the experimental phases of false-memory research, when the experimenter debriefs participants at the end of the study. Debriefing after a suggestive procedure might thus be one method for experimentally creating and exploring nonbelieved memories, and was thus the focus of the present study.

Testing the effects of debriefing on beliefs and memories is important for two reasons. First, as we have outlined, debriefing could provide a way to create and thus to systematically investigate nonbelieved memories. Second, and more broadly, it raises the practical question of whether participants in false-memory experiments tend to leave those studies with the effects of the induction fully reversed by the experimenter's debriefing. In other words, does debriefing successfully ‘undo’ participants' false memories, or does it simply ‘undo’ their beliefs, leaving nonbelieved memories intact?

In the present study we used Nash and colleagues' doctored-video procedure to induce false memories in participants [9], [10]: participants saw doctored video clips that purported to ‘prove’ they had performed actions that they did not truly perform. A few hours later they were fully debriefed, after which we re-assessed their beliefs and memories to see whether their false memories were ‘still there’. At this point we also assessed the characteristics of participants' beliefs and memories. Using this doctored-video procedure has at least two benefits for our purposes: First, the procedure has been shown to induce high rates of strongly-held false beliefs and memories, as compared to other false-memory paradigms such as the imagination inflation procedure that tend to induce significant but small confidence increases [11], [12]. Second, in Nash et al.'s studies, many participants made informal remarks after debriefing that they could ‘still remember’ performing the false actions that were suggested. This observation gives credence to the hypothesis that debriefing after the doctored-video induction could leave nonbelieved memories behind.

The results of the study confirm the prediction that the debriefing in a false memory study leaves behind memory-like experiences for recent events. These are probably mental images that to a large extent feel like genuine memories, even though the belief in those mental images is substantially reduced by the debriefing. It also revealed that Nash et al.'s paradigm produces a smaller number of non-believed memories before the debriefing, thus creating clear memories for actions that participants are not very certain to have performed. These data confirm that memories and beliefs are independently-occurring constructs and as such can be manipulated independently.

## Materials and Methods

### Ethical approval

This research has been approved by the Ethics Committee at the Department of Psychology, University of Hull, UK.

### Participants and Design

Twenty participants (18 females, 2 males) completed all sessions of the study; their ages ranged from 18–54, (*M* = 24.15, *SD* = 9.13). Participants who studied psychology were compensated with course credits; non-psychology students participated voluntarily without compensation. The study had a within-subjects design, with critical action type (performed, fake, new) and session (Session 2 pre-debriefing, Session 3 post-debriefing) as the manipulated variables.

### Materials and procedure

We selected 42 of the simple actions from [10] for use in the various stages of this study. From these, we selected six actions to be critical actions (*clap your hands, click your fingers, rub the table, salute, cover your face with your hands*, and *flex your arm*). The critical actions were selected on the basis that they were neither highly memorable nor unmemorable, based on the ratings collected in the Nash et al. study. These six actions were randomly divided into three pairs that were assigned—counterbalanced across participants—as the performed, fake and new critical actions. Performed critical actions were genuinely performed by participants in Session 1; fake actions were not performed, but doctored evidence presented in Session 2 suggested that they were indeed performed; new critical actions were neither performed nor suggested, but appeared only in the belief and memory questionnaires in Sessions 2 and 3, and were used as a control.

#### Session 1

The procedure used here was modelled after Nash et al.'s [9], [10] procedure. Participants were greeted by a researcher, and informed that the study was investigating people's ability to mimic others' actions. They were told that their task would involve observing the researcher performing a series of actions, and then copying the actions themselves. They were also informed that they would be video-recorded as they completed this task. After gaining consent, the researcher and participant sat at a table facing each other, and with the video-camera directed toward them both. The researcher started filming the session, and began by performing a simple action for 12 sec. After this period, the participant was then required to copy the action they had seen for a further 12 sec. Next the researcher performed a second action, and this ‘observe—copy’ process continued until both the researcher and the participant had performed 26 actions, including the 2 critical actions that had been assigned as ‘performed actions’. The 24 non-critical actions were performed in a single randomized order in all participants' sessions, as these were essentially fillers. The critical performed actions were always performed in the 9th and 17th position of the sequence.

After completing all 26 actions, the participant was thanked and reminded to return for Session 2. Once the participant had left the room, the researcher returned to the table and filmed himself performing the two critical actions that had been assigned as fake actions. The researcher performed each of these for 12 sec while seated in the same position as he had sat while the participant was present.

#### Preparing the video-sequences

**Figure 1:**
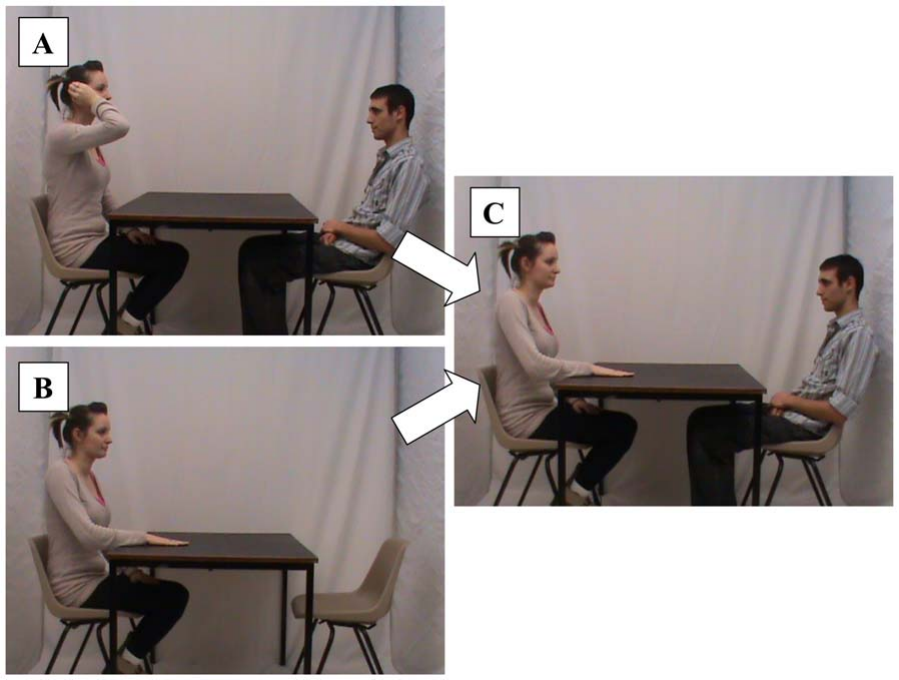
Video manipulation. (A) Real clip. (B) Fake action. (C) Doctored composite of (A) and (B).

Following Session 1, we used Adobe After Effects software to doctor two clips from the video-recording. As in Nash et al. [9], [10], and as depicted in Figure 1, each doctored clip was created by digitally combining two genuine clips: one that showed the researcher performing a critical action *after* the participant had left the room, and one that showed the participant passively observing a different action. The images from these clips were combined to produce composites that seemed to prove that the participant had in fact observed the two fake actions. Because participants also performed all of the actions they observed, these clips were therefore designed to persuade participants that they had also performed these two actions. Next, we used Adobe Premier Pro to embed these doctored clips into a longer sequence of clips taken from the genuine recording. The full sequence comprised clips of the two fake actions, the two performed critical actions, and eight other non-critical performed actions as fillers; all participants saw the same eight filler actions. All 12 clips were 10-sec in length, and separated by 5-sec pauses during which the screen was blank; thus the full video-sequence lasted just under 3 min. We did not want the critical actions to be highly salient in the video-sequence; the performed critical actions were thus placed in positions 3 and 7 and fake actions in positions 5 and 10 of the video sequence.

#### Session 2

Participants returned for Session 2 two days after Session 1. In this session participants were shown their 3-min video-sequence twice through. To ensure participants paid attention to the actions in the video, on the first viewing they were asked to note down how many times they thought they performed each action in a week. On the second viewing, participants were asked to name each action. Participants next completed a 5-min filler task (solving anagrams), after which they completed two questionnaires that asked whether they *believed* and *remembered* that they performed various specific actions during Session 1. Participants completed the memory questionnaire first: this questionnaire listed 28 actions including 22 non-critical fillers (of which 10 were performed in Session 1 and 12 were new) and the 6 critical actions. For each action, participants used an 8-point scale to rate their memory, in response to the question “How strongly do you *remember* performing this action in Session 1?”. Following the memory questionnaire, participants completed the belief questionnaire, which comprised the same 28 actions in a different order. Here, participants again used an 8-point scale to answer the question “How strongly do you *believe* you performed this action in Session 1?”. In both questionnaires, a rating of ‘8’ signified a strong belief or memory. Our initial piloting showed that participants understood the distinction between belief and memory better when the memory questionnaire was administered first, and so we did not counterbalance this ordering. Doing so might have negated a possible confound insofar as people's belief ratings might have been influenced by their memory ratings; however, for the purpose of this exploratory study, we decided it preferable that participants were fully able to understand the conceptual difference. After completing these questionnaires, participants were again thanked and reminded to return for Session 3.

#### Session 3

Participants returned for Session 3 approximately 4 hours after Session 2. In this session, we explained to participants that some of the video-clips they saw in Session 2 had been doctored, and we told them which clips were the fakes. For each of the six critical actions, participants were then asked to provide new belief and memory ratings using the same scales as in Session 2. Finally, participants completed a questionnaire probing the phenomenological characteristics of their memories. For each of the six critical actions, they were asked to rate 25 memory characteristics on 7-point scales (see Materials S1).

## Results

In the following section we present our findings in four stages. First we examine the data measuring participants' beliefs and memories for critical actions in Session 2, and we look for evidence of nonbelieved memories among these reports. Second, we conduct the same analyses on the comparable data from Session 3. Third, we look at the changes in participants' ratings between Session 2 and Session 3. Fourth, we analyze the phenomenology data collected in Session 3. Although we also analysed the data with two repeated measures factorial ANOVAs including Session (Session 2 vs. Session 3) as a within-subjects variable, for ease of interpretation we report the outcomes of separate analyses of Session 2 data, Session 3 data, and change-scores. The results of the overall ANOVAs were wholly consistent with those reported here, with large Critical Action Type x Session interaction effects both for Belief ratings, *F*(2, 38) = 20.00, *p*<.001, η^2^_p_ = .51, and Memory ratings, *F* (2, 38) = 8.73, *p* = .001, η^2^_p_ = .32.

### Beliefs and memories, pre-debriefing

As a manipulation check, we were first interested to find whether our doctored videos led participants to believe or remember they performed the fake actions. To this end, we examined participants' action ratings from Session 2; these are represented in the first column of data in Table 1. A one-way repeated measures ANOVA revealed significant differences in belief ratings across critical action types, *F*(2, 18) = 73.43, *p*<.001, η^2^_p_ = .89. Follow-up paired sample *t*-tests showed that performed critical actions were rated significantly higher than both new critical actions, *t*(19) = 10.76, *p*<.001, *d_z_* = 2.41, and fake actions, *t*(19) = 2.32, *p* = .03, *d_z_* = 0.52. Importantly, fake actions were rated significantly higher than new critical actions *t*(19) = 8.99, *p*<.001, *d_z_* = 2.01, which shows that our doctored videos had the intended effect on beliefs. The same pattern of results held for memory ratings: there were significant differences across critical action types *F*(2, 18) = 62.50, *p*<.001, η^2^_p_ = .87. Performed critical actions were rated significantly higher than both new critical actions, *t*(19) = 11.37, *p*<.001, *d_z_* = 2.54, and fake actions, *t*(19) = 3.37, *p*<.01, *d_z_* = 0.75, but fake actions were rated significantly higher than new critical actions, *t*(19) = 7.23, *p*<.001, *d_z_* = 1.62. Together these findings support those of Nash et al. [9], [10] and show that the doctored-video procedure was effective at distorting participants' beliefs and memories for their actions.

**Table 1:** Mean belief and memory ratings assigned to critical actions before and after debriefing.

		Before debriefing		After debriefing		Change (After – Before)	
		*M*	*SD*	*M*	*SD*	*M*	*SD*
**Belief**	Performed actions	7.15	1.34	6.48	1.40	−0.68	1.13
	Fake actions	5.93	1.83	2.63	1.90	−3.30	2.84
	New actions	2.20	1.27	2.83	2.05	+0.63	1.78
**Memory**	Performed actions	7.30	1.13	6.48	1.63	−0.83	1.26
	Fake actions	5.78	1.92	3.73	1.87	−2.05	2.43
	New actions	2.33	1.48	2.55	1.83	+0.23	1.96

We also assessed whether participants reported any nonbelieved memories in this session. Recall that in Scoboria et al. [6], participants gave memory ratings that were higher than their belief ratings on just 4.3% of occasions. In Session 2 of the present study, memory ratings were higher than belief ratings on 14.2% of occasions (10% of performed critical actions; 15% of fake actions; 17.5% of new critical actions). This frequency of nonbelieved memories is considerably higher than Scoboria et al.'s figure.

Random variations in participants' ratings might account for many of the nonbelieved memories when assessed in this way, particularly because unlike Scoboria et al. we administered the belief and memory questionnaires separately. For this reason we also examined our data with more stringent criteria. First, we classified responses as nonbelieved memories only if the memory rating was at least 2 scale-points higher than belief rating. This pattern held on 10.8% of occasions (7.5% of performed critical actions; 12.5% of fake actions; 12.5% of new critical actions). When the difference was required to be 3 or more scale-points, the overall rate was 5.8% (5% performed, 5% fake, 7.5% new).

### Beliefs and memories, post-debriefing

At the start of Session 3, we asked participants to guess what the aim of the study was. Only one participant guessed a hypothesis involving false memory or doctored videos; this participant was removed from analysis and replaced with another participant.

We now turn to examining whether debriefing influenced people's beliefs and memories, and whether it created any nonbelieved memories. To this end, we began by examining participants' belief and memory ratings from Session 3, after they had been debriefed. These results are reported in the middle column of data in Table 1. A one-way repeated measures ANOVA on the belief ratings again revealed significant differences across action types, *F*(2, 18) = 38.56, *p*<.001, η^2^_p_ = .81. Performed critical actions were rated higher than both new critical actions, *t*(19) = 7.42, *p*<.001, *d_z_* = 1.66, and fake actions, *t*(19) = 8.16, *p*<.001, *d_z_* = 1.83, but this time fake actions were no longer rated higher than new critical actions in terms of belief, *t*(19) = −0.42, *p* = .68, *d_z_* = 0.09. In other words, the debriefing appeared to undo the effect of the doctored video-clips on participants' beliefs. Results were partly different for memory ratings. An ANOVA revealed significant differences in memory ratings across critical action types, *F*(2, 18) = 41.29, *p*<.001, η^2^_p_ = .82. As before, performed critical actions were rated higher than new critical actions, *t*(19) = 9.30, *p*<.001, *d_z_* = 2.08, and fake actions, *t*(19) = 5.09, *p*<.001, *d_z_* = 1.14, but unlike the pattern with the belief ratings, memory ratings for fake actions remained significantly higher than those for new critical actions, *t*(19) = 2.70, *p* = .01, *d_z_* = 0.60. That is to say, the debriefing did not undermine participants' memories of fake actions to the same extent as it undermined their beliefs.

These analyses suggest that debriefing might have created some additional nonbelieved memories. To assess whether this was the case, as for Session 2 we examined participants' Session 3 ratings to see how often their memory ratings exceeded their belief ratings by at least one scale-point: the criterion used in [1]. Overall, this occurred for 26.7% of critical actions (20.0% of performed critical actions; 42.5% of fake actions; 17.5% of new critical actions). As compared to the Session 2 data, following debriefing there were significantly more nonbelieved memories of fake actions, *z* = 2.30, *p* = .02. The same was not true of performed critical actions, *z* = 1.16, *p* = .25, or new critical actions, *z* = 0.00, *p* = 1.00. Indeed, as Table 1 illustrates, after debriefing the mean memory ratings were significantly *greater* than the belief ratings only for fake actions, *t*(19) = 3.51, *p*<.01, *d_z_* = 0.79; in all other conditions the belief and memory ratings did not significantly differ (for all contrasts, *t*<1.1, *p*>.29, *d_z_*<.25).

When the more stringent criterion to measure nonbelieved memories (memory minus belief ≥2 scale-points) was used, a lower number of nonbelieved memories was obtained (14.2% overall), but the decrease was mostly for performed (5%) and new critical actions (7.5%). For fake actions memory ratings were at least two points higher than belief ratings on 30% of occasions. A similar pattern was found when the even more stringent criterion of ≥3 scale-points was used, with 10.8% of nonbelieved memories overall. Still 25% of the fake actions met this criterion, but only 5% of the performed critical actions and 2.5% of the new critical actions. It is therefore clear that our false memory induction and debriefing procedure substantially increased the incidence of nonbelieved memories even when a highly stringent classification criterion was used.

### Change-scores

To explore our findings in more depth, we calculated change-scores by subtracting participants' belief and memory ratings given at Session 2 from their ratings given at Session 3. These change-scores are shown in the third column of data in Table 1, and provide a measure of the effect of debriefing on beliefs and memories. One-sample *t*-tests showed that with regard to performed critical actions, the change-scores were significantly below zero for both the belief, *t*(19) = −2.93, *p*<.01, *d* = 0.60, and memory measures, *t*(19) = −2.68, *p* = .02, *d* = 0.66. These change-scores for performed critical actions give us an indication of how much deflation in ratings between Sessions 2 and 3 might plausibly be attributed to simple weakening of memory-strength and confidence across the time delay. Change-scores for fake actions were also significantly below zero, (Belief, *t*(19) = −5.19, *p*<.001, *d* = 1.16, Memory, *t*(19) = −3.78, *p* = .001, *d* = 0.84), but were also significantly greater in magnitude than those for performed critical actions (both *t*s>2.5, both *p*s<.05, both *d*s>0.56). These change-scores therefore show that both beliefs *and* memories for fake actions were undermined by debriefing, although the effect on belief was significantly greater than the effect on memory. The change-scores for new critical actions did not differ significantly from zero (Belief, *t*(19) = 0.51, *p* = .61, *d* = 0.11; Memory, *t*(19) = 1.57, *p* = .13, *d* = 0.35).

### Phenomenological characteristics

The final element of our analysis was to look at the characteristics of participants' beliefs and memories. Recall that at the end of Session 3, participants rated all six critical actions in terms of 25 memory characteristics. For each of these 25 characteristics we computed a one-way ANOVA, with critical action type as the repeated measures factor. After making a Bonferroni correction (α = .05/25 = .002), these analyses revealed significant differences on 9 of the 25 memory characteristics, including visual detail, feelings, and the experience of re-living. However, follow-up *t*-tests with Bonferroni corrections revealed that all of these effects were driven by memory characteristics for performed critical actions being clearer than for fake and new critical actions. In contrast, there were no significant differences between the characteristics of memories for fake actions and new critical actions.

**Figure 2:**
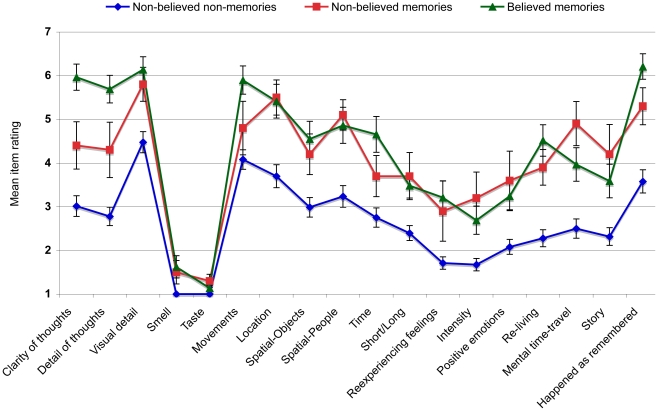
Phenomenological characteristics that differed between nonbelieved memories, believed memories and nonbelieved-nonmemories.

To assess whether nonbelieved memories differ in characteristics from other types of belief and memory phenomena, we collapsed the Session 3 data across critical action types, and categorised all 120 critical actions (6 actions×20 participants) as either a nonbelieved non-memory (*n* = 76), believed non-memory (*n* = 5), nonbelieved memory (*n* = 10), or believed memory (*n* = 29). Responses were classified as ‘beliefs’ whenever participants gave belief ratings of 7 or 8, and also as ‘memories’ whenever they gave memory ratings of 7 or 8. Thus instead of defining nonbelieved memories as before in terms of the size of the difference between memory and beliefs scores, here a nonbelieved memory is defined specifically as a memory rated as 7 or 8, accompanied by a belief rated 6 or below. We conducted a series of one-way ANOVAs to compare the characteristics of different response-types; however, we excluded believed non-memories from this analysis due to their low frequency. As represented in Figure 2, our ANOVAs revealed significant differences (α = .05/25 = .002) on 18 of the 25 memory characteristics. We were particularly interested in whether nonbelieved memories differed from nonbelieved non-memories (i.e., comparing nonbelieved events with vs. without an accompanying memory), and from believed memories (i.e., comparing memories with vs. without an accompanying belief). Follow-up *t*-tests revealed that nonbelieved memories were rated as significantly richer than nonbelieved non-memories on 12 of the 18 measures that had been significant overall; three of these were significant at the Bonferroni-adjusted level (α = 0.05/18 = 0.0028): memory for location, *t*(17.88) = 3.76, *p* = .001, *d* = 0.98, spatial arrangement of people, *t*(19.82) = 4.36, *p*<.001, *d* = 1.09, and feeling of mental time-travel, *t*(84) = 3.75, *p*<.001, *d* = 1.35. In contrast, only two characteristics—clarity of thought and details of thought (both *t*s<2.61, both *d*s<0.75)—differed between nonbelieved memories and believed memories, and neither remained significant after a Bonferroni-adjustment. These findings—along with a visual inspection of Figure 2—broadly support those of Mazzoni et al. [1], insofar as they show that nonbelieved memories share many more similarities with believed memories than they do with non-remembered events.

## Discussion

Ours is the first study to our knowledge to systematically examine whether the effects of a powerful false-memory induction are ‘undone’ when participants are debriefed, or whether nonbelieved memories are left behind. The data provide new evidence—the first experimental evidence—for the proposal that the occurrence of beliefs and memories can be independent. Further building on the work of Mazzoni et al. [1] in which participants described nonbelieved memories of childhood experiences, the present study also represents the first empirical demonstration of nonbelieved memories of *recent* experiences.

Confirming previous results [9], the manipulation used to induce false memories was highly effective. Many false memories for fake actions were obtained: 68% of memory ratings were above the scale-midpoint, and a high percentage (58%) were in the high confidence range (i.e., Memory ≥7). The debriefing manipulation we used significantly increased participants' tendency to rate their memories for fake actions as stronger than their belief in those actions, a response pattern that previous studies have shown to occur only rarely [6]. This was true even with our most stringent criterion: For 25% of fake actions, memory ratings were at least three points above belief ratings, whereas this was true for just 4% of other critical actions. Indeed, after debriefing, participants' mean memory ratings for fake actions were significantly higher than their mean belief ratings for those actions (and also higher than their mean memory ratings for new critical actions). These results suggest that after debriefing participants were left with some residual memory-like content for the fake actions, that they did not believe to be grounded in genuine experience.

The study of the dissociation between beliefs and memories stems from research on the effects of suggestion, in which often the creation of false beliefs has not been accompanied by false memories [13]. This dissociation is important not only in false memories (e.g., [14]), but also in episodic autobiographical memory more generally [3], [6], and for understanding some clinical conditions [5]. Previously, the distinction has been conceived as a partial dissociation, in which memories are nested within belief [6]. Here we have shown experimentally that the two are theoretically independent, as the same manipulation affects differently beliefs and memories. This leads to having believed memories (what are usually called episodic memories); believed but not remembered events; nonbelieved memories, and nonbelieved and not remembered events. It is well established that procedures that create false memories often increase beliefs more easily than memories [13], [15]. Similarly, this study shows that procedures that aim at deleting false memories have a greater effect on the belief than the memory. In other words, beliefs seem to be in general more malleable than memories. We are unaware of any theoretical reason to expect gender effects in terms of this relative malleability; however, the low proportion of male participants in the present study and the exclusively student sample are limitations to the generalizability of these conclusions that should be addressed in further studies.

Using the 2×2 classification system we explored the phenomenological characteristics of participants' beliefs and memories, to help understand what the word ‘memory’ might refer to. In the current study believed and nonbelieved memories (combined across all action types) did not differ on any measure that reflected a recollective experience. This indicates that nonbelieved memories still maintain a strong sense of recollection (see also [1]), while differing on non-recollective characteristics involving thoughts (*details of thought* and *clarity of thought*). In contrast, several recollective characteristics differed between nonbelieved memories and nonbelieved non-memories; thus, memory, as opposed to belief, could be conceived as recollection. We note, however, that one key experience not assessed here is familiarity, which in some previous studies has been shown to affect belief judgments [16], [17], and in other studies has been shown also to affect memory judgments [18], [19]. Future studies should independently manipulate in the same procedure familiarity and recollection and assess how they relate separately to belief.

One important question that remains unanswered by the present study relates to the nature of the independence between belief and memory. Is belief a necessary precursor to memory that can nevertheless be removed afterwards, like scaffolding on a new building? Or, alternatively, can memories form completely in the absence of belief? One might reason that the former hypothesis would be true: a memory-like image that develops in the absence of belief would feasibly be attributed to a dream or to imagination. Indeed, it might be that a belief itself can cause mental images to be attributed to memory; belief could thus be conceptualised as a form of source-monitoring cue in its own right, a conceptualisation that fits with existing theoretical accounts of metacognitive processes in autobiographical memory in which the strength of the belief affects memorial processes. The idea that belief can function as a monitoring cue in its own right also is in line with other attributional models in which non-memorial information (such as perceptual fluency) affects the ‘old/new’ decision in recognition tasks [19], [20]. Nevertheless, evidence against this interpretation—and in favour of the latter hypothesis—is that many of the nonbelieved memories in our study were not a product of our suggestive doctored videos and debriefing: participants occasionally reported nonbelieved memories for fake actions in Session 2, as well as for performed and new actions in Sessions 2 and 3. Thus here nonbelieved (sometimes false) memories have occurred spontaneously and independently of the experimental manipulation. These observations raise the intriguing possibility that memories might indeed sometimes form in the absence of belief. This can occur only if beliefs and memories are the product of different mechanisms.

Finally, our findings have broader implications for memory distortion research. To the extent that debriefing might not always completely ‘undo’ the effects of a suggestive manipulation, we might question the ethics of inducing false memories in experimental participants. Is it ethical for participants to leave research labs with remnants of nonbelieved false memory content in the forefront of their minds? A sensible approach to answering this question might be to consider whether the memories would likely be consequential. For example, it is conceivable that a person who ceased believing in a traumatic experience might nevertheless continue to be traumatised by intrusive mental images experienced as memories. We suggest that for most false-memory paradigms and study designs, this is highly unlikely to pose an ethical problem. Nevertheless, how participants might feel about any residual memory content should be an important question for researchers to consider when planning studies.

## Supporting Information

Material S1 **The twenty five memory characteristics rated for each of the six critical actions. Ratings were on 7-point scales.**
(DOC)
