# Supplementary material for: Creating Non-Believed Memories for Recent Autobiographical Events
Source: PLoS One. 2012 Mar 9;7(3):e32998. doi: 10.1371/journal.pone.0032998 (PMC3302900; doi:10.1371/journal.pone.0032998)
Supplement: Material S1 — The twenty five memory characteristics rated for each of the six critical actions. Ratings were on 7-point scales. (DOC) [file pone.0032998.s001.doc]

*Material S1.* The twenty five memory characteristics rated for each of the six critical actions. Ratings were on 7-point scales.

**Phenomenology Questionnaire**

1. The clarity of my thinking about this action is: (1 = dim; 7 = sharp/clear)
2. The details of my thinking for this action are: (1 = sketchy; 7 = very detailed)
3. When I think about this action it involves visual details: (1 = not at all; 7 = very much)
4. When I think about this action it involves sound: (1 = not at all; 7 = very much)
5. When I think about this action it involves smell: (1 = not at all; 7 = very much)
6. When I think about this action it involves touch: (1 = not at all; 7 = very much)
7. When I think about this action it involves taste: (1 = not at all; 7 = very much)
8. When you think of this action, the movements are: (1 = vague; 7 = clear/distinct)
9. When I think about this action, the location where the action takes place is: (1 = vague; 7 = clear/distinct)
10. When I think about this action, the relative spatial arrangement of objects is: (1 = vague; 7 = clear/distinct)
11. When I think about this action, the relative spatial arrangement of people in my memory is: (1 = vague; 7 = clear/distinct)
12. When I think about this action, the time when the action takes place is: (1 = vague; 7 = clear/distinct)
13. The action seems (short, long): (1 = short; 7 = long)
14. I experience how I felt at the time when the action took place: (1 = not at all; 7 = very much)
15. When I think about this action, the feelings contained in it are: (1 = negative; 7 = positive)
16. When I think about this action, the feelings contained in it are: (1 = not intense; 7 = very intense)
17. As I am thinking about the action now, my feelings are: (1 = not intense; 7 = very intense)
18. The strength of my positive emotions for this action are: (1 = none; 7 = very intense)
19. The strength of my negative emotions for this action are: (1 = none; 7 = very intense)
20. As I think about the action, I feel as though I am re-living the action: (1 = not at all; 7 = very much)
21. As I think about the action, I feel that I travel back to the time when it happened, that I am a subject in it, rather than an outside observer tied to the present: (1 = not at all; 7 = very much)
22. As I think about the action, it comes to me in words or in pictures as a coherent story or episode and not as isolated scenes, facts or thoughts: (1 = not at all; 7 = coherent story)
23. As I think about the action, it is connected with other actions: (1 = not at all; 7 = very much)
24. I have thought or talked about this action before: (1 = not at all; 7 = very much)
25. I believe the action really occurred in the way I remember it: (1 = not at all; 7 = very much)
